# Brain endothelial cells promote breast cancer cell extravasation to the brain via EGFR-DOCK4-RAC1 signalling

**DOI:** 10.1038/s42003-024-06200-x

**Published:** 2024-05-18

**Authors:** Chiara Galloni, Teklu Egnuni, Safoura Zahed Mohajerani, Jiaqi Ye, Sibylle Mittnacht, Valerie Speirs, Mihaela Lorger, Georgia Mavria

**Affiliations:** 1 Leeds Institute of Medical Research, University of Leeds, Leeds, UK; 2 UCL Cancer Institute, University College London, London, UK; 3 Institute of Medical Sciences, University of Aberdeen, Aberdeen, UK; 4 Present Address: Sheffield Institute for Nucleic Acids (SInFoNiA) and School of Biosciences, University of Sheffield, Sheffield, UK; 5 Present Address: Leeds Centre for Disease Models, University of Leeds, Leeds, UK

**Keywords:** Cancer, Breast cancer

## Abstract

The role of endothelial cells in promoting cancer cell extravasation to the brain during the interaction of cancer cells with the vasculature is not well characterised. We show that brain endothelial cells activate EGFR signalling in triple-negative breast cancer cells with propensity to metastasise to the brain. This activation is dependent on soluble factors secreted by brain endothelial cells, and occurs via the RAC1 GEF DOCK4, which is required for breast cancer cell extravasation to the brain in vivo. Knockdown of DOCK4 inhibits breast cancer cell entrance to the brain without affecting cancer cell survival or growth. Defective extravasation is associated with loss of elongated morphology preceding intercalation into brain endothelium. We also show that brain endothelial cells promote paracrine stimulation of mesenchymal-like morphology of breast cancer cells via DOCK4, DOCK9, RAC1 and CDC42. This stimulation is accompanied by EGFR activation necessary for brain metastatic breast cancer cell elongation which can be reversed by the EGFR inhibitor Afatinib. Our findings suggest that brain endothelial cells promote metastasis through activation of cell signalling that renders breast cancer cells competent for extravasation. This represents a paradigm of brain endothelial cells influencing the signalling and metastatic competency of breast cancer cells.

## Abstract (web summary)

Brain endothelial cells activate EGFR signaling in triple-negative breast cancer cells, promoting brain metastasis. DOCK4 is crucial for cancer cell extravasation to the brain, with EGFR activation driving cell elongation reversed by Afatinib.

## Introduction

Cancer metastasis is the leading cause of cancer-related deaths with brain metastases being particularly devastating as they result in extreme morbidity and mortality^1,2^. Breast cancer is commonly associated with brain metastases, particularly in the case of triple-negative breast cancer (TNBC) defined by the absence of estrogen receptor (ER), progesterone receptor (PR), and human epidermal growth factor 2 (HER2). Brain metastasis occurs in approximately one-third of TNBC patients^3^. Following haematogenous dissemination during the metastatic process, circulating breast cancer cells must arrest within the luminal space of brain capillaries, and then extravasate across the endothelium into the brain parenchyma^4,5^. This process is enabled by the interaction of cancer cells with the brain endothelium, which is partially characterised. Some molecules that promote breast cancer cell adhesion to the endothelium, and mediate the passage to the brain parenchyma have been identified including ST6GALNAC5, COX2, MMP1, HBEGF and VLA-4 (α4β1)^6–9^. The interaction results in changes in cancer cell morphology, disassembly of endothelial junctions, and alterations within the brain parenchyma that collectively promote successful extravasation^4,5,10^.

Breast cancer often exhibits Epidermal Growth Factor Receptor (EGFR) overexpression, and studies have reported that TNBC patients also exhibit EGFR overexpression with a prevalence rate that varies depending on the patient cohort^11,12^. EGFR overexpression has been associated with poor prognosis, and an increased rate of metastasis in TNBC patients and has been proposed as a potential therapeutic target, with some patients demonstrating a response to EGFR inhibitors in clinical trials^12^. EGFR signalling is known to activate multiple downstream signalling pathways involved in cancer cell survival, proliferation and migration^13^. Furthermore, during brain metastasis EGFR plays a crucial role in the crossing of the brain endothelium by breast cancer cells, and in promoting their survival and growth in the brain^6,14^. However, it is unclear whether the interaction of breast cancer cells with endothelial cells influences EGFR activation, and which downstream signalling molecules are involved. The Rho family GTPases play a critical role in cancer progression and metastasis, partly due to their ability to regulate the actin cytoskeleton of metastasising cancer cells, thus influencing their morphology, polarity and migratory potential^15,16^. Their activation downstream of growth factor signalling is largely driven by guanine nucleotide exchange factors (GEFs) responsible for facilitating the exchange of GDP for GTP^15,16^. In addition to regulating cancer cell invasion, Rho proteins are also involved in the disruption of the endothelial barrier function, promoting extravasation of cancer cells into the underlying tissue^17^.

In this study, we demonstrate that the Dedicator of Cytokinesis 4 (DOCK4), a GEF implicated in RAC1 activation downstream of EGFR signalling^18^, is necessary for induction of a cancer cell mesenchymal-like morphology compatible with intercalation of breast cancer cancer cells into brain endothelium in vitro, and brain metastatic extravasation in vivo. We show that the pathway is activated by factors secreted by brain endothelial cells (BEC) in the absence of direct BEC-cancer cell interaction. BEC-conditioned media promote EGFR activation, breast cancer cell elongated morphology and filopodial protrusions; and knockdown of either DOCK4 or RAC1 reverses cancer cell elongation without affecting filopodia. Elongated morphology also requires the GEF Dedicator of Cytokinesis 9 (DOCK9) and Cell Division Control protein 42 (CDC42). Hence, BEC promote metastatic extravasation through activation of EGFR signalling in breast cancer cells that requires DOCK4, DOCK9, RAC1 and CDC42, rendering them competent for extravasation. Understanding the mechanisms of brain extravasation has the potential to identify strategies to prevent the process of metastasis of breast cancer cells to the brain.

## Results

### DOCK4 is required for breast cancer cell extravasation to the brain in vivo

**Fig. 1:**
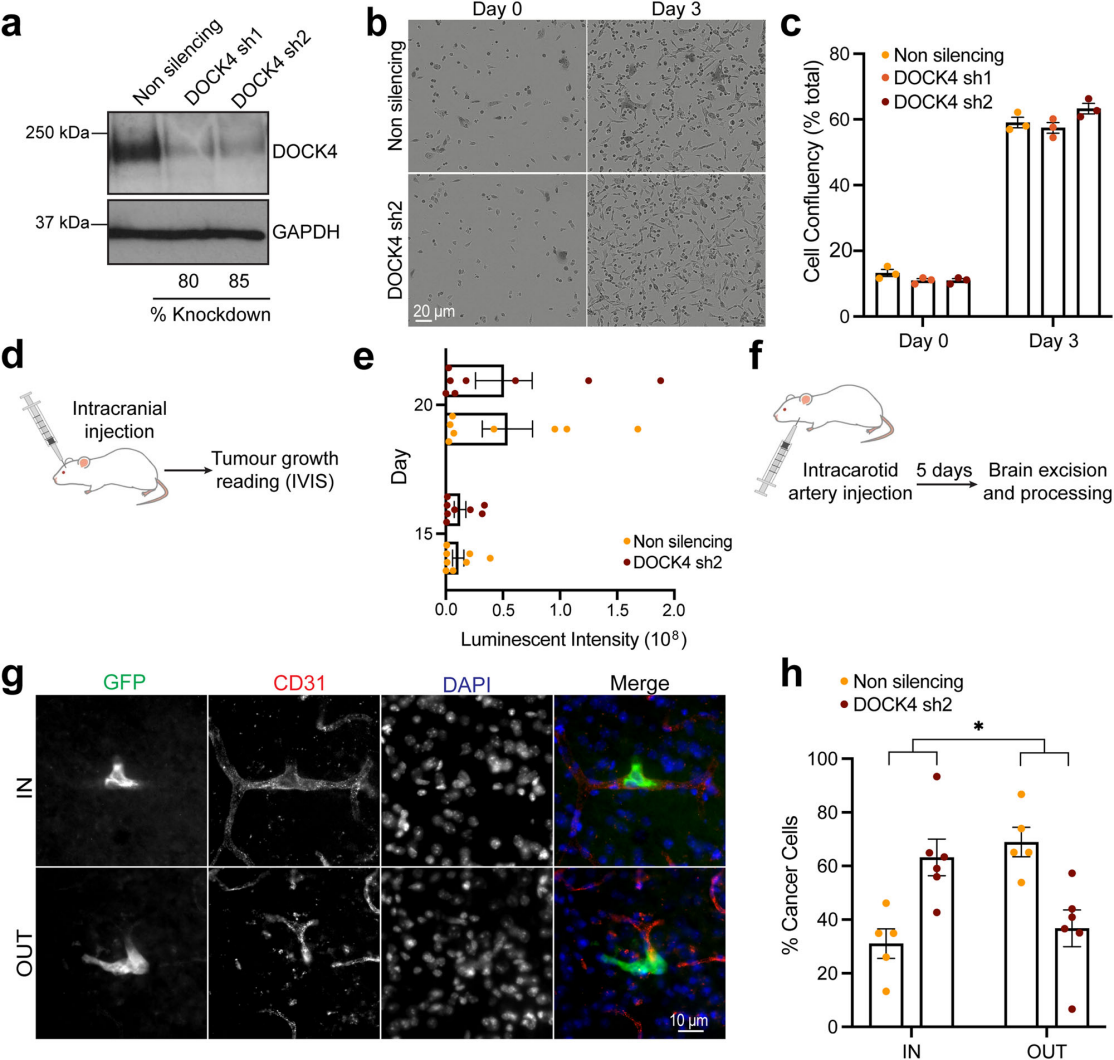
DOCK4 knockdown inhibits breast cancer cell extravasation to the brain. **a** Immunoblot analysis of MDA-MB-231/Brain cells following DOCK4 stable depletion (DOCK4 sh1, DOCK4 sh2). **b** Still phase images from Supplementary Movie 1 of real-time assessment of MDA-MB-231/Brain cell growth upon stable DOCK4 depletion (DOCK4 sh2). Scale bar = 20 µm. **c** Graph shows changes in confluency of MDA-MB-231/Brain cells following DOCK4 depletion (DOCK4 sh1, DOCK4 sh2). Error bars represent SEM from 3 biological replicates. **d** Schematic depicts intracranial implantation of MDA-MB-231/Brain cells stably expressing control (Non silencing) or DOCK4 (sh2) shRNAs followed by monitoring of tumour growth by bioluminescence imaging (IVIS). **e** Graph shows the increase of bioluminescence signal at days 15 and 20 post-intracranial injection. Error bars represent SEM for *N* = 10 mice for each condition. **f** Schematic depicts injection of MDA-MB-231/Brain cells into the carotid artery of mice followed by isolation of brains after 5 days. **g** Confocal images show MDA-MB-231/Brain cells expressing GFP identified within (IN) or outside (OUT) CD31 positive blood vessels in cryo-sections of excised brains. Scale bar = 10 µm. **h** Graph shows the percentage of cancer cells located inside (IN) versus outside (OUT) blood vessels 5 days post-intracarotid artery injection of MDA-MB-231/Brain cells. Error bars represent SEM from 2 independent experiments in which 100 cells were scored per mouse brain; *N* = 6 and *N* = 5 brains analysed from mice injected with cells stably expressing control (Non silencing), or DOCK4 (sh2) shRNAs respectively. *P < 0.05 by two-tailed Student’s *t* test.

Previous studies have shown an association of DOCK4 and the metastatic phenotype of cancer cells^19,20^. In this study, we investigated the role of DOCK4 in the process of breast cancer cell extravasation and the growth of brain metastases. We first showed that stable knockdown of DOCK4 did not affect the in vitro proliferation of brain homing breast cancer cells^21^ (MDA-MB-231/Brain) (Fig. 1a–c and Supplementary Movie 1). We then implanted these cells intracranially in mice and assessed tumour growth using bioluminescence imaging (IVIS) over a 20-day period (Fig. 1d). Analysis revealed that DOCK4 does not play a role in the growth of breast cancer cells in the brain, as cells lacking DOCK4 are as efficient as control cells in tumour growth in the brain parenchyma (Fig. 1e). Next, we investigated whether DOCK4 is important for extravasation by injecting DOCK4 depleted cells into the internal carotid artery of mice and determining the percentage of breast cancer cells that localised inside or outside the brain blood vessels 5-days post intracarotid injection (Fig. 1f). We found that 5 days post-injection, the majority of DOCK4 depleted cells (>60%) were still trapped inside the mouse brain capillaries, whereas in comparison, approximately 70% of control cells had already extravasated (Fig. 1g, h). These data suggest that while DOCK4 does not play a role in the growth of breast tumours colonising the brain, it is required for effective extravasation of cancer cells cells from the bloodstream into the brain parenchyma.

### EGF and brain endothelial cell-secreted factors promote cancer cell elongation and endothelial intercalation through DOCK4 and RAC1

**Fig. 2:**
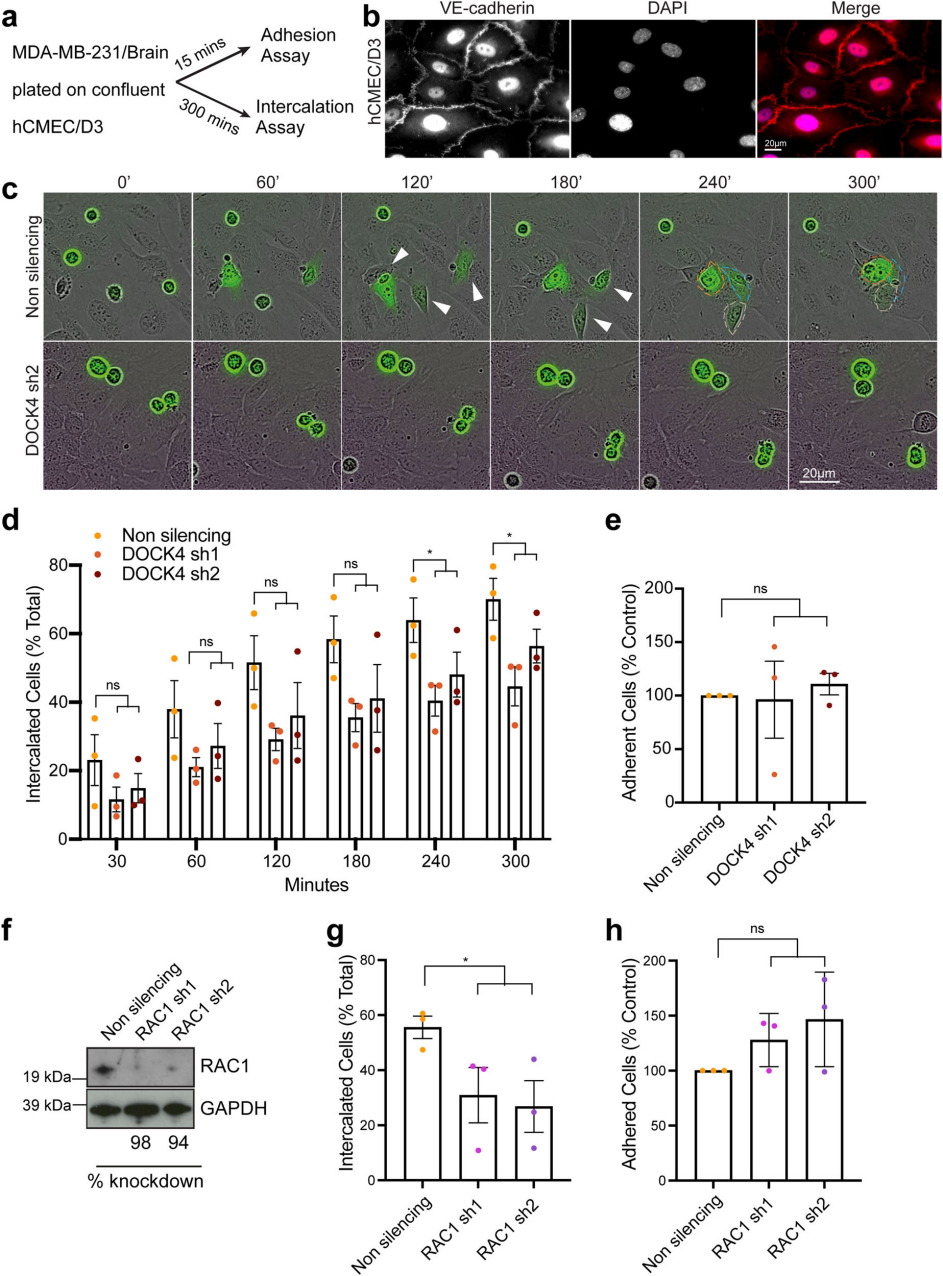
Knockdown of DOCK4 or RAC1 blocks breast cancer cell intercalation into brain endothelial cells. **a** Schematic depicts parallel in vitro adhesion and intercalation assays following seeding of MDA-MB-231/Brain cells onto confluent human brain endothelial cells (hCMEC/D3). Adhesion was determined 15 min and intercalation 5 h post-seeding. **b** Immunofluorescence images of confluent hCMEC/D3 monolayer prior to seeding MDA-MB-231/Brain cells. Adherens junctions (VE-cadherin) were visualised. Scale bar = 20 µm. **c** Still phase images from timelapse movies (see Supplementary Movie 2) showing MDA-MB-231/Brain cells (green) transduced with either control (Non silencing) or DOCK4 (sh2) shRNAs seeded onto confluent hCMEC/D3. White arrowheads indicate elongated cells prior intercalation. Scale bar = 20 µm. **d** Graph shows quantification of MDA-MB-231/Brain cells intercalated into hCMEC/D3 (% total) from timelapse movies. **e** Graph shows adhesion of MDA-MB-231/Brain cells 15 min post-seeding onto confluent hCMEC/D3. Data expressed as percentage of control (non silencing). **f** Immunoblot analysis of MDA-MB-231/Brain cells following RAC1 stable depletion (RAC1 sh1, RAC1 sh2). **g** Graph shows quantification of MDA-MB-231/Brain cells intercalated into hCMEC/D3 (% total) from timelapse movies at the timepoint when ≥50% of control (non silencing) cells intercalated into hCMEC/D3. **h** Graph shows adhesion of MDA-MB-231/Brain 15 min post-seeding onto confluent hCMEC/D3. Data are expressed as the percentage of control (non silencing). **d**, **e**, **g**, **h** Error bars represent SEM from *N* = 3 independent experiments in which ≥100 cells were analysed from ≥9 movies per condition in 3 technical replicates per experiment. *P < 0.05 by two-tailed Student’s *t* test.

One key step of the extravasation process is represented by the crossing of the endothelial layer termed transendothelial migration (TEM)^22^. Prior to this, cancer cells adhere and insert into the endothelial monolayer via a process termed intercalation^22,23^. We investigated the ability of breast cancer cells to both adhere to or intercalate through confluent brain endothelial cells (BEC) (hCMEC/D3)^24^ (Fig. 2a, b). In vitro intercalation assays revealed that DOCK4 depleted breast cancer cells display a significant delay in intercalation compared to control cells (Fig. 2c, d and Supplementary Movie 2); whereas adhesion assays performed in parallel showed that cancer cells lacking DOCK4 are as efficient as control cells in adhering to BEC (Fig. 2e). Hence, we conclude that DOCK4 regulates the extravasation of breast cancer cells into the brain via controlling the stage of intercalation. As DOCK4 is a RAC1 GEF^25^, and RAC1 has been implicated in TEM^26^, we investigated the role of RAC1 in the adhesion and intercalation processes of breast cancer cells. RAC1-depleted cells (Fig. 2f) were seeded onto confluent BEC, and adhesion and intercalation assays were performed. The experiments showed that RAC1 is required for intercalation but not for adhesion of breast cancer cells onto BEC (Fig. 2g, h).

**Fig. 3:**
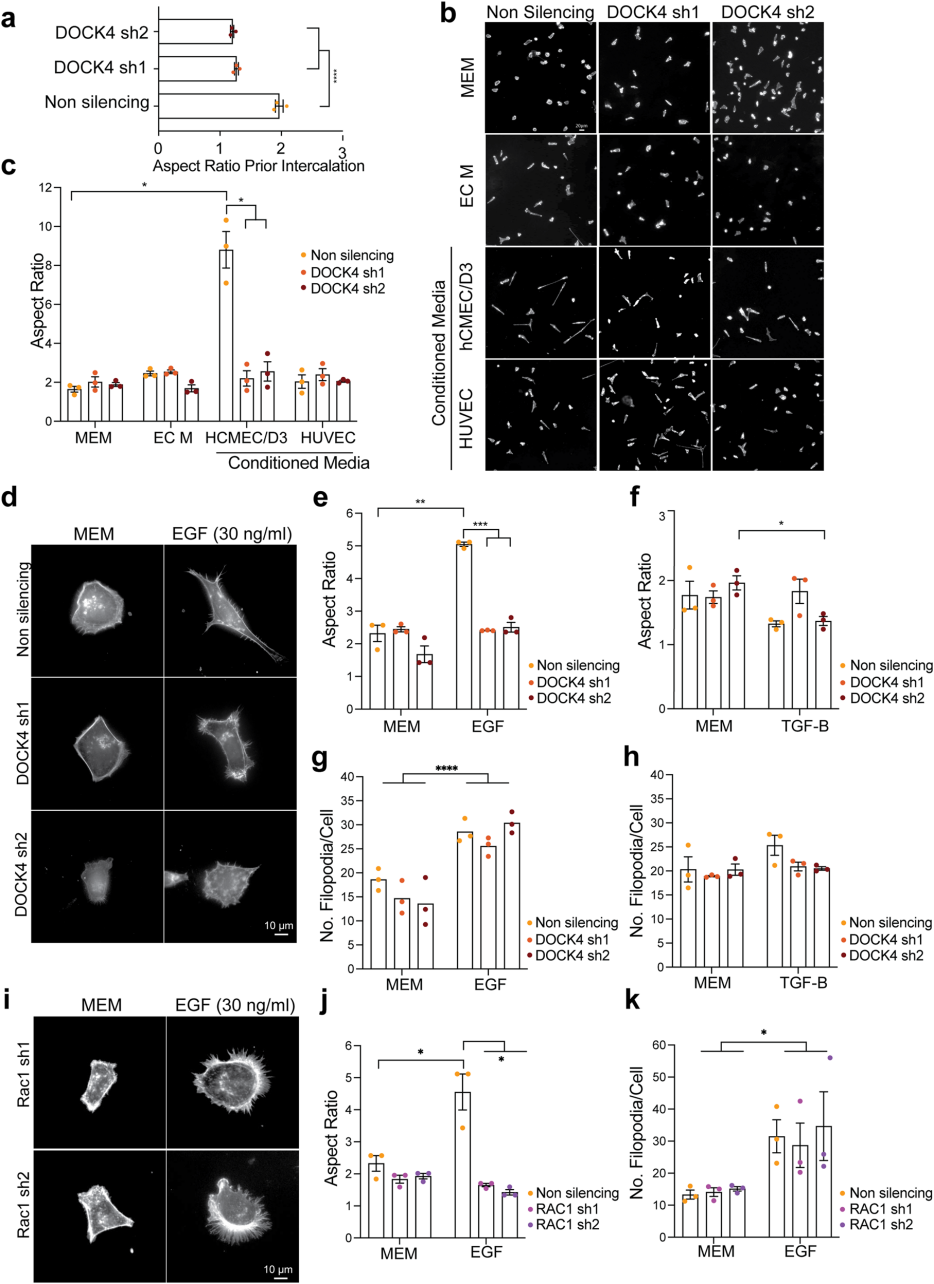
DOCK4 and RAC1 control breast cancer cell elongation driven by brain endothelial cells and EGF. **a** Graph shows elongation (Aspect Ratio, length/width) of MDA-MB-231/Brain control (non silencing), or with DOCK4 depletion (sh1 and sh2) prior to intercalation 4 h post-seeding onto hCMEC/D3. Error bars represent SEM from 2 independent experiments in Fig. 2c in which 240 cells were analysed from ≥6 movies per condition in 3 technical replicates per experiment. ****P < 0.0001 by two-tailed Student’s *t* test. **b** Immunofluorescence images of MDA-MB-231/Brain cells upon serum-starvation followed by 4 h stimulation with control Minimal Essential Medium (MEM), Endothelial Cell Medium (EC M), or conditioned media harvested from hCMEC/D3 or HUVEC. **c** Graph shows the Aspect Ratio of cells shown in **b**. Error bars represent SEM from *N* = 3 independent experiments in which 80 cells were analysed per condition. **d** Immunofluorescence images of MDA-MB-231/Brain cells control (non silencing) or with stable DOCK4 depletion (DOCK4 sh1, DOCK4 sh2) upon serum starvation followed by 4 h stimulation with 30 ng/ml Epidermal Growth Factor (EGF). **e**, **f** Graphs show the Aspect Ratio of MDA-MB-231/Brain in **d** upon 4 h stimulation with MEM, 30 ng/ml EGF (**e**), or 50 ng/ml Transforming Growth Factor β (TGFβ). Error bars represent SEM from *N* = 3 independent experiments in which a total of 80 cells were analysed per condition. **g**, **h** Graphs show the number of filopodia of MDA-MB-231/Brain cells in **d**. Error bars represent SEM from *N* = 3 independent experiments in which 90 cells were analysed per condition. **i** Immunofluorescence images of MDA-MB-231/Brain stably depleted of RAC1 (sh1 and sh2) upon serum starvation followed by 4 h stimulation with 30 ng/ml EGF. Graphs show the Aspect Ratio (**j**) and number of filopodia (**k**) of cells shown in **i**. Error bars represent SEM from 3 independent experiments in which a total of 81 (**j**) or 90 (**k**) cells were analysed per condition. MDA-MB-231/Brain cells in **b**, **d**, **i** were labelled with Alexa Fluor 594 Phalloidin to visualise the actin cytoskeleton. Scale Bars = 10 µm. *P < 0.05; **P < 0.01; ***P < 0.001, ****P < 0.0001 by two-tailed Student’s *t* test.

We investigated the cellular mechanism by which DOCK4 mediates breast cancer cell intercalation. Fröse and co-workers previously demonstrated that a mesenchymal-like elongated morphology facilitates cancer cell extravasation during breast cancer metastasis to the lungs^27^. Thus, we explored the potential role of a mesenchymal-like elongated morphology in brain extravasation. To measure the elongation of breast cancer cells seeded onto confluent BEC, we calculated the aspect ratio, which is the ratio between the cell length and width. Our results showed that control cells adopt an elongated morphology prior to intercalation (Fig. 2b), while DOCK4-depleted cells were significantly less elongated compared to controls 240 min post-seeding onto the BEC monolayer (Fig. 3a). Next, we investigated whether BEC promote elongation via activation of a signalling cascade that involves DOCK4. Breast cancer cells stimulated with conditioned media harvested from BEC (hCMEC/D3) showed an increased aspect ratio compared to cells stimulated with endothelial cell media (EC M) and this phenotype was blocked by DOCK4 depletion (Fig. 3b, c). Furthermore, breast cancer cells did not elongate in response to conditioned media harvested from non-brain endothelial cells (HUVEC) (Fig. 3b, c). These data demonstrate that BEC promote elongation of breast cancer cells via DOCK4, and that activation does not require cell-cell contact but is mediated via secreted factors.

We explored whether endothelial-derived factors such as EGF and TGFβ (Transforming growth factor β) promote the elongation of triple-negative breast cancer cells (Fig. 3d–f). Previous studies have shown that both EGF and TGFβ can be secreted by endothelial cells and induce the acquisition of a mesenchymal-like phenotype in breast cancer cells^28,29^. Our results showed that upon EGF stimulation, DOCK4-depleted cells do not elongate to the same extent as control cells (Fig. 3d, e). Additionally, breast cancer cells do not elongate in response to TGFβ stimulation (Fig. 3f), suggesting that EGF, but not TGFβ, stimulates the acquisition of an elongated phenotype via DOCK4. As previous studies have shown that increased filopodia number and length upon EGF and TGFβ stimulation promote invasiveness of cancer cells^30,31^ and DOCK4/RAC1 control lateral filopodia formation^25^, we quantified the number of filopodia on breast cancer cells cells upon EGF and TGFβ stimulation and their potential dependence on DOCK4/RAC1 (Fig. 3d, g, h). Our results showed that control and DOCK4-depleted cancer cells almost double their filopodia in response to EGF treatment (Fig. 3d, g), whereas upon TGFβ stimulation, the number of filopodia remained unchanged in both control and DOCK4-depleted cells (Fig. 3h). These data indicate that EGF stimulates filopodia formation in via a signalling cascade that does not require DOCK4. We next investigated if RAC1 is essential for breast cancer cell elongation. Our results showed that cells lacking RAC1 do not elongate as control cells in response to EGF (Fig. 3i, j). Quantification of filopodia showed that upon EGF stimulation, both control and RAC1 depleted cells almost double their filopodia (Fig. 3k). These experiments suggest that DOCK4 and RAC1 promote the intercalation of breast cancer cells into the brain endothelium via regulating cell elongation but not filopodial protrusions in response to endothelial-derived growth factors, such as EGF. Taken together, our data, along with previous findings showing the activation of RAC1 downstream of EGFR via DOCK4^18^ suggest that BEC-secreted factors, including EGF, activate EGFR and RAC1 via DOCK4 to promote an elongated phenotype and extravasation competency of tripe-negative breast cancer cells.

### Brain endothelial cell-secreted factors activate EGFR to promote breast cancer cell elongation

**Fig. 4:**
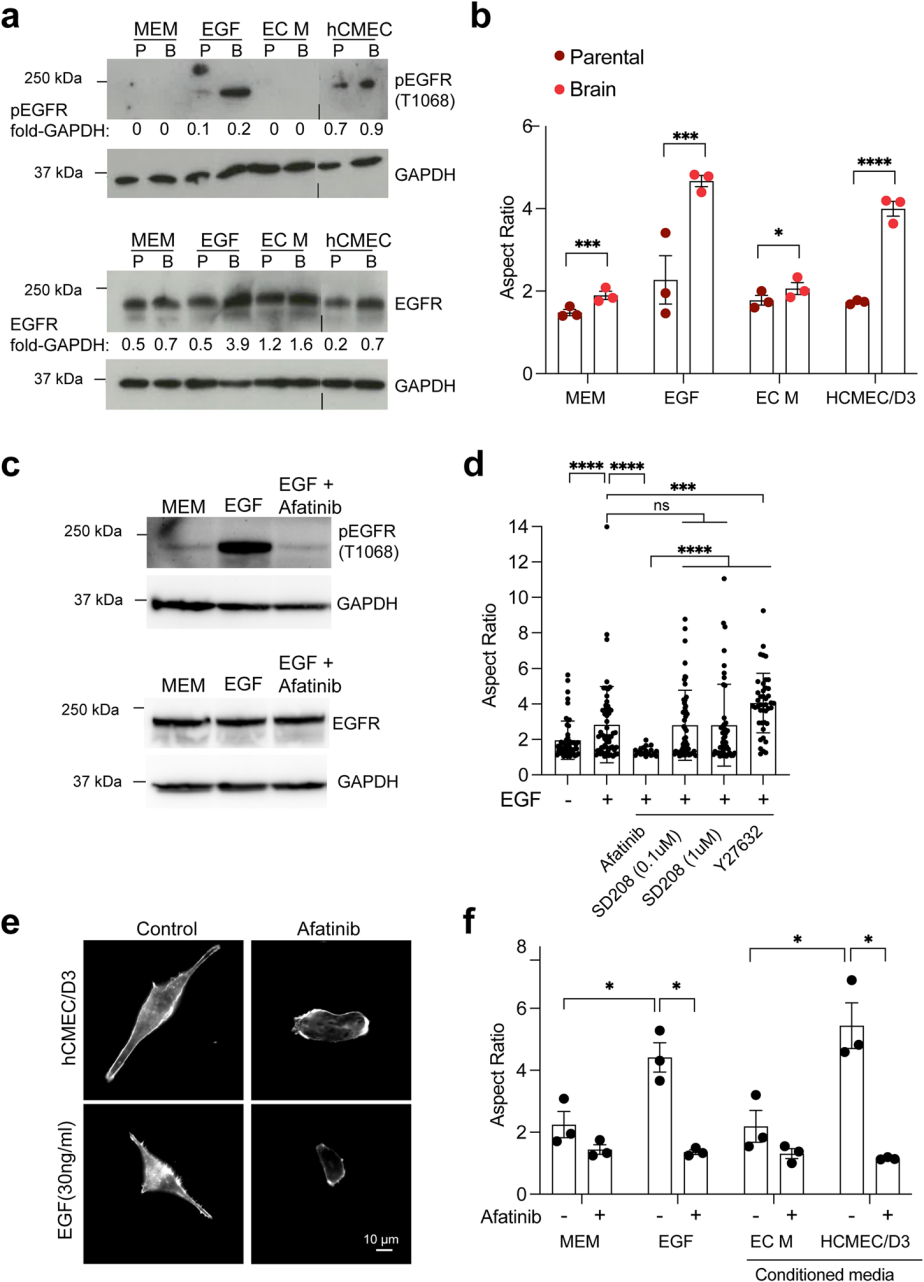
EGFR is activated by brain endothelial cell factors and is necessary for brain metastatic breast cancer cell elongation. **a** Immunoblot shows EGFR activation (pEGFR) by hCMEC/D3-conditioned media or EGF in MD-MB-231/Parental (P) and MDA-MB-231/Brain (B) cells upon serum starvation followed by stimulation with EGF or hCMEC/D3-conditioned media for 4 h. **b** Quantification of aspect ratio of MDA-MB-231/Brain MDA-MB-231/Parental cells upon serum starvation followed by stimulation with EGF or hCMEC/D3-conditioned media for 4 h. Error bars represent SEM from *N* = 3 independent experiments in which ≥90 cells were analysed per condition in 3 technical replicates per experiment. **c** Immunoblot shows EGFR activation by EGF in MDA-MB-231/Brain cells upon serum starvation followed by stimulation with EGF for 4 h in the absence or presence of Afatinib (5 μM). **d** Quantification of aspect ratio of MDA-MB-231/Brain upon serum starvation followed by stimulation with EGF in the absence or presence of Afatinib (5 μM), SD208 (0.1 μM or 1 μM), or Y27632 (10 μM). Error bars represent SEM from a pilot experiment in which a minimum of 18 cells were analysed per condition. **e** EGFR inhibition with Afatinib blocks elongation of MDA-MB-231/Brain driven by EGF or hCMEC/D3-conditioned media. **f** Quantification of aspect ratio of MDA-MB-231/Brain upon serum starvation followed by stimulation with EGF or hCMEC/D3-conditioned media for 4 h with control Minimal Essential Medium (MEM), EGF, Endothelial Cell Medium (EC M), or hCMEC/D3-conditioned media in the absence or presence of Afatinib. Error bars represent SEM from three independent experiments in which a minimum of 40 cells were analysed per condition. *P < 0.05; ***P < 0.001; ****P < 0.0001 by two-tailed Students’ *t* test.

We examined whether BEC-secreted factors promote EGFR activation and whether there were differences between parental and brain homing MB-MDA-231 cancer cells that may account for the ability of MB-MDA-231/Brain cells to respond to BEC-secreted factors via cellular elongation (Fig. 4a, b). Previous studies have shown that EGFR is necessary for the crossing of an endothelial barrier by brain-homing breast cancer cells in vitro and for brain metastasis in vivo^6^, and elevated EGFR levels have been reported in triple-negative breast cancer cells with propensity to metastasise^11,13^. Our results showed that stimulation of breast cancer cells with BEC (hCMEC/D3)-conditioned media but not control media (MEM or EC M), stimulated EGFR phosphorylation to levels similar to those of EGF stimulation (Fig. 4a). Furthermore, there was increased EGFR prosphorylation in MDA-MB-231/Brain compared to parental MDA-MB-231 cells, which correlated with increased levels of total EGFR (Fig. 4a). To further investigate the role of EGFR in the response of breast cancer cells to BEC-secreted factors, we treated the cells with Afatinib, an inhibitor of the ErbB family kinases, including EGFR^32^. First we confirmed that Afatinib treatment blocks EGFR activation in MDA-MB-231/Brain cells (Fig. 4c). Our results showed that treatment with Afatinib, but not with the unrelated inhibitors SD208 (an inhibitor of the TGFβ receptor ALK5) or Y27632 (a Rho-kinase inhibitor), reversed the stimulation of elongated morphology by BEC-secreted factors and EGF (Fig. 4d–f). These data support the notion that BEC-secreted factors activate EGFR, which then promotes a DOCK4-dependent elongated morphology compatible with intercalation and metastatic brain extravasation.

### DOCK9 and CDC42 control breast cancer cell elongation and endothelial intercalation

**Fig. 5:**
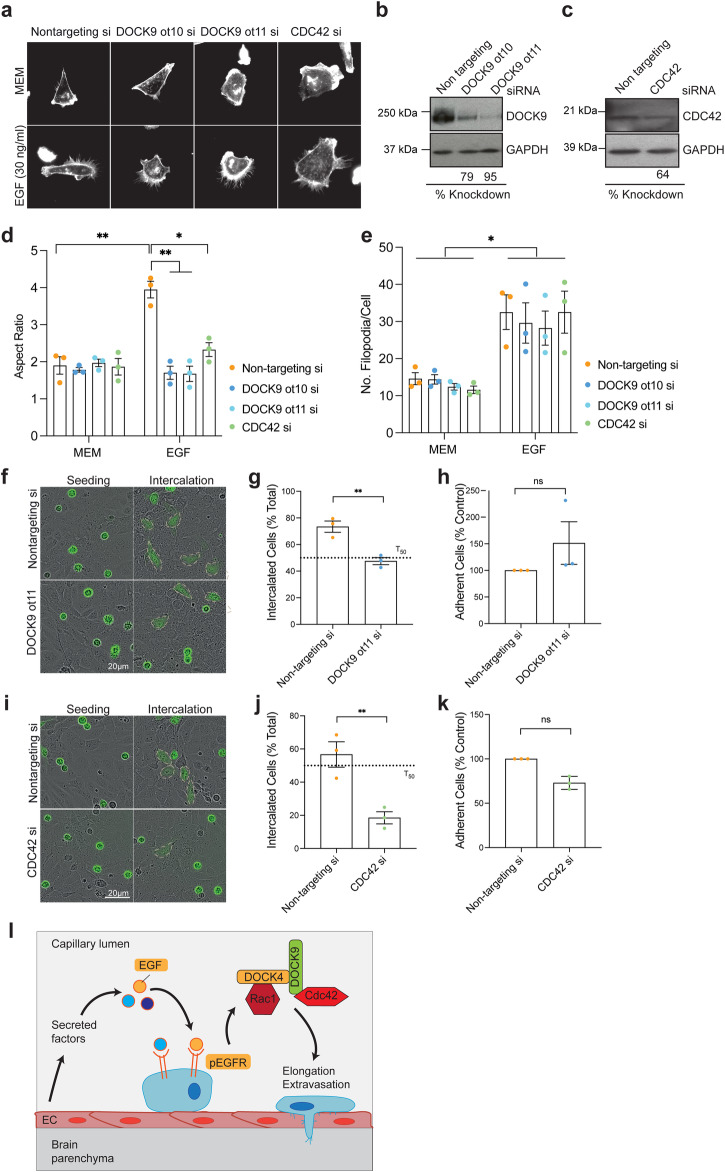
DOCK9 and CDC42 control breast cancer elongation and intercalation into brain endothelial cell monolayers. **a** Immunofluorescence images of MDA-MB-231/Brain cells transfected with on-target siRNAs, DOCK9 (ot10 and ot11), or CDC42 (smartpool) upon serum starvation followed by 4 h stimulation with EGF (30 ng/ml). Cells were labelled with Alexa Fluor 594 Phalloidin to visualise the actin cytoskeleton. Scale Bar = 10 µm. **b** Immunoblot analysis of MDA-MB-231/Brain cells transfected with control (non targeting) or DOCK9 siRNAs (ot11 and ot10). **c** Immunoblot analysis of MDA-MB-231/Brain cells transfected with control (non targeting) or CDC42 siRNA (smartpool). Graphs show quantification of elongation (Aspect Ratio) (**d**) and number of filopodia (**e**) of MDA-MB-231/Brain cells in **a**. Error bars represent SEM from 3 independent experiments in which a total of 81 (**d**) and 90 (**e**) cells were analysed per condition. *P < 0.05; **P < 0.001 by two-tailed Student’s *t* test. **f** Still phase images showing intercalation of MDA-MB-231/Brain cells stably expressing Lifeact-GFP transfected with control (non targeting) or DOCK9 siRNAs (ot11 and ot10) seeded onto confluent hCMEC/D3. Dotted lines mark intercalated cells. Scale Bar = 20 µm. Graphs show the percentage of MDA-MB-231 cells, control (non targeting) or with DOCK9 knockdown (ot11) intercalated into hCMEC/D3 at T_50_ (**g**), or adhered 15 min post-seeding (**h**). **i** Still phase images showing intercalation of MDA-MB-231/Brain cells stably expressing Lifeact-GFP transfected with with control (non targeting) or CDC42 siRNAs (smartpool) seeded onto confluent hCMEC/D3. Dotted lines mark intercalated cells. Scale Bar = 20 µm. Graphs show the percentage of MDA-MB-231 cells control (non targeting) or with CDC42 knockdown intercalated into hCMEC/D3 at T_50_ (**j**), or adhered 15 minutes post-seeding (**k**). **g**, **h**, **j**, **k** error bars represent SEM from *N* = 3 independent experiments in which ≥100 cells were analysed from ≥9 movies per condition in 3 technical replicates per experiment. Note the elongation of breast cancer cells interacting with hCMEC/D3 is blocked with knockdown of DOCK9 (**f**) or CDC42 (**i**). **l** Model of breast cancer cell extravasation into the brain parenchyma promoted by brain endothelial cell activation of EGFR signalling. Brain endothelial cell-derived factors and EGF promote an elongated morphology competent for brain extravasation driven by EGFR signalling and RAC1/ CDC42 activation via GEFs DOCK4 and DOCK9. *P < 0.05; **P < 0.01 by two-tailed Student’s *t* test.

Small interfering RNA screens targeting Rho GTPases in human prostate cancer cells identified CDC42 as a critical regulator of cancer cell-endothelial cell interaction and intercalation^33^. In addition, we have previously shown that DOCK4 can interact with DOCK9, a CDC42 GEF, and this signalling cascade controls lateral filopodia and tubule formation in ECs^25^. Hence, we investigated the role of DOCK9 and CDC42 in elongation and intercalation of breast cancer cells (Fig. 5). Elongation assays showed that, like DOCK4-depleted cells (Fig. 3d, e), cells lacking either DOCK9 or CDC42 did not elongate upon EGF stimulation as control cells (Fig. 5a–d), suggesting that DOCK9 and CDC42, together with DOCK4 and RAC1, are essential for the elongation and subsequent intercalation of breast cancer cells. In addition, as previously observed for DOCK4 and RAC1 (Fig. 3g, k), DOCK9 and CDC42 are not required for filopodia formation in response to EGF stimulation, as both control and DOCK9 or CDC42 depleted cells doubled the number of filopodia upon EGF treatment (Fig. 5e). Intercalation and adhesion assays showed that breast cancer cells depleted of DOCK9 adhere to BEC as efficiently as control cells; however, they show a significant delay in intercalation compared to control cells at the time point where ≥ 50% control cells have intercalated (T_50_) (Fig. 5f–h). Similar adhesion and intercalation phenotypes were observed upon CDC42 knockdown (Fig. 5i–k). Although cells with CDC42 knockdown adhere to the brain endothelium as efficiently as control cells, they intercalate much slower (Fig. 5i–k). These data suggest that DOCK9 and CDC42 play an essential role in promoting the intercalation of breast cancer cells into brain endothelial cells. Altogether, our data suggest that the brain endothelium and EGF contribute to extravasation by promoting DOCK4/RAC1- and DOCK9/CDC42-mediated elongation and intercalation of breast cancer cells.

## Discussion

In this study we demonstrate how factors secreted by brain endothelial cells (BEC) activate EGFR signalling via DOCK4 to promote an elongated phenotype in breast cancer cells, thereby facilitating their successful extravasation to the brain. The activation of RAC1 downstream of EGFR also supports this conclusion^18^. In line with this, using xenograft mouse models, we show that DOCK4 plays a key role in promoting the extravasation of cancer cells through the brain capillaries in vivo, while it does not affect tumour growth and progression in the brain. Consistent with these findings, previous studies have shown that EGFR ligands, such as EREG and HBEGF, stimulate breast cancer cell transendothelial migration, while EGFR inhibition promotes survival following introduction of brain homing breast cancer cells into the circulation in vivo^6^. The results of our study offer insight into how endothelial cells can influence the metastatic capability of cancer cells. By stimulating breast cancer cells with growth factors released by endothelial cells, such as EGF or TGFβ, we found that EGF, but not TGFβ, can lead to breast cancer cell elongation compatible with extravasation. These results were surprising because both growth factors have previously been shown to promote elongation and invasion of breast cancer cells in vivo and in vitro in spheroid and matrigel invasion assays^34,35^. However, in those studies, the elongated morphology was additionally driven by Ras^35^, or EGF drove downstream TGFβ signalling in MCF-7 cells^34^. Thus, while TGFβ is a mediator of epithelial-mesenchymal transition (EMT) conferring cancer cell plasticity, it does not drive the elongated, invasive morphology in triple-negative breast cancer cells.

During metastatic extravasation cancer cells undergo alterations of their actin cytoskeleton, allowing them to squeeze through openings of the endothelial barrier^10^. Our study shows that adoption of elongated morphology, driven by endothelial factors, is tightly associated with the process of extravasation. This is supported by previous studies investigating morphological changes in cancer cells during the metastatic process. Kienast and co-workers used intracranial windows and two-photon microscopy to follow metastasis and observed cancer cells adopting an elongated shape along blood vessel lumens^4^, a step followed by dynamic extensions and retractions of extravascular protrusions during the active process of extravasation^4^. In breast cancer lung metastasis, upregulation of the surface protein podocalyxin promotes transition of cancer cells from a non-polarised, rounded shape to an elongated, invasive morphology necessary for crossing microvascular networks in vitro and tissue colonisation in vivo^27^. In brain metastasis, breast cancer cell elongation along the vessel lumens precedes the development of endothelial blebs that isolate invading cells from the circulation, facilitating extravasation^36^.

Because of their function in regulating the cytoskeleton and actomyosin contractility several Rho proteins and their regulators have been implicated in the process of cell invasion and metastasis in vitro and in vivo, but less is known about their role during the process of cancer cell extravasation. Our study shows that factors secreted by brain endothelial cells, including EGF, promote both cell elongation and upregulation of filopodial protrusions associated with the metastatic phenotype^30^. Blocking the elongated morphology but not filopodial protrusions by knockdown of the RAC GEF DOCK4 inhibits cancer cell intercalation in vitro, and brain extravasation in vivo. Knockdown of the DOCK4 interaction partner DOCK9, or CDC42, had similar effects in blocking elongated morphology and intercalation, while filopodial protrusions remained unaffected. This suggests that in breast cancer cells responding to factors secreted by brain endothelial cells, EGF controls filopodia via a pathway that does not involve CDC42, and filopodia may be dispensible for elongation and invasiveness during metastatic extravasation^37,38^; moreover it has been demonstrated previously that filopodia formation may be independent of CDC42^38^. Interestingly, mechanical signals conveyed by physical constraints within small capillaries can also change the morphology of cancer cells to an elongated phenotype, leading to changes in gene expression compatible with invasiveness and extravasation^39,40^. Therefore, both paracrine signalling from the endothelium and mechanical deformation in response to physical constraints can alter the shape and behaviour of cancer cells during extravasation.

Survival of metastasising cancer cells in the microcirculation is influenced by their ability to withstand fluid shear stress and biomechanical constriction forces, a capability attributed to upregulation of actomyosin contractility^41,42^. Interestingly, fluid forces remodel the vasculature to generate regions permissive for the arrest of cancer cells surviving in the circulation^43^. Our study suggests that EGFR and DOCK4-mediated RAC1 activation in response to secreted brain endothelial factors oppose this contractile phenotype, allowing cancer cells to cross the endothelium and propel themselves into the brain parenchyma (Fig. 5l). In support of this notion, patient-derived cancer cells alter their phenotype while in the circulation, exhibiting a predominantly round morphology after release from the primary tumour, and mesenchymal shape after crossing the capillary bed^44^. We postulate that following extravasation, cessation of EGFR-DOCK4-RAC signalling by yet unidentified factors must suppress the invasive phenotype, for initiation of micrometastases at sites of activation of the endothelium^45^. Previous research has identified DOCK4 as a potential biomarker for breast cancer bone metastasis^20^. Hence, common molecular mechanisms involving DOCK4/RAC1 may promote extravasation of breast cancer cells to the other metastatic sites. This is supported by the study of Klotz and co-workers, who utilised cell lines derived from patient circulating tumour cells and found partial sharing of mediators of metastasis to the brain and bone^46^. In the case of lung metastasis in breast cancer, ezrin plays a crucial role by linking podocalyxin at the plasma membrane to the underlying actin cytoskeleton, which establishes dorsal cortical polarity of breast cancer cells enabling their transition to the mesenchymal-like, extravasation-competent shape. Ezrin is a susbtrate for EGFR and is involved in RAC1 activation through interaction with RhoGDIs (guanine nucleotide dissociation inhibitors) and RAC1 localisation to specific regions of the plasma membrane^47^. Therefore, it may participate in EGFR signalling via DOCK4 during the process of brain extravasation. In future studies, a more comprehensive understading of the signalling pathway can be achieved by conducting knockdown and overexpression experiments in conjunction with GTPase activation assays^25^. This approach will help elucidate the potential reciprocal regulation of RAC1, CDC42 and their activating GEFs in brain homing breast cancer cells. Moreover, it will be imperative to elucidate the in vivo roles of RAC1, DOCK9 and CDC42 in the process of brain metastatic extravasation.

In conclusion, inhibiting brain extravasation could potentially extend the survival of patients with metastatic disease. Our study highlights the role of endothelial cells in influencing breast cancer cells’ ability to extravasate to the brain and identifies molecules involved in this process. Studies on the pathways of metastatic extravasation have potential to lead to the development of strategies to prevent brain metastasis. It is worth noting that brain metastasis may have not occurred in many patients with metastastic disease to other organs; and cancer cells in the circulation may repopulate the brain following standard of care treatment of metastatic disease.

## Methods

### Cell culture and antibodies

MDA-MB-231/Parental and MDA-MB-231/Brain cells^21^ were cultured in Eagle’s minimum essential medium supplemented with L- Glutamine, vitamin mix, non essential amino acids, sodium pyruvate, penicillin-streptomycin, and 10% foetal calf serum (FCS). MDA-MB-231/Brain cells were stably transduced with lentiviral vector expressing LifeAct-GFP to allow their detection by immunofluorescence. For in vivo imaging MDA-MB-231/Brain was stably transduced with Firefly luciferase (F-luc)^5^. hCMEC/D3 cells^24^ (a kind gift from Prof Graham Cook) and HUVEC (TCS CellWorks) were cultured in Human Large Vessel Endothelial Cell Growth Medium (TCS CellWorks). All cells were cultured at 37 °C with 5% CO_2_. Antibodies were sourced as follows: Abcam, anti-GFP (ab2090, 1:1000); BD Biosciences, anti-CD31 (clone MEC 13.3, 1:50); Bethyl Laboratories, anti-DOCK4 (A302-263A, 1:1,000); Cell signalling, anti-EGFR (D38B1, 1:500), anti-pEGFR (D7A5, 1:500); Millipore, anti-RAC1 (clone 23A8, 1:1,000); Proteintech, anti-GAPDH (60004, 1:1,000), anti-DOCK9 (18987; 1:1,000); Santa Cruz, anti-CDC42 (sc-8401, 1:50); Thermo Fisher Scientific, Alexa Fluor secondary antibodies 488 and 594 (1:200).

### Transfection and lentiviral transduction

MDA-MB-231/Brain cells (1.5 ×10^5^ per well) were seeded onto 6-well plates (Corning) and transfected with 10 nM siRNA using Lipofectamine 2000 RNAiMax (Invitrogen) and Opti-MEM I Reduced Serum Medium (Gibco) according to the manufacturer’s recommendations. Assays were performed 48 h after transfection. For stable knockdown MDA-MB-231/Brain cells expressing shRNAs and EGFP, or empty vector EGFP control were generated by lentiviral infection in the presence of polybrene (8 μg ml^−1^), and EGFP expressing cells were FACS sorted and expanded for use in assays. Lentiviral vectors (pGIPZ) were obtained from Open Biosystems. The sequences of DOCK9 and CDC42 siRNAs, and DOCK4 shRNAs were as previously listed^25^.

### Western blotting

Cells were washed once in PBS before being lysed with lysis buffer (50 mM Tris-HCl pH 7.4, 100 mM NaCl, 5 mM MgCl2, 1% NP-40, 10% Glycerol, 1 mM DTT and EDTA-free complete protease inhibitor). The protein samples were span down at 13,000 rpm at 4 °C prior to determine protein concentration using BSA protein assay kit (Thermo Fisher Scientific) and following the manufacturer instructions. NuPAGE LDS Sample Buffer (Thermo Fisher Scientific) with the addition of 50 mM DTT was then added to the cell lysates and the samples were incubated at 98 °C for 1 min. For EGFR and pEGFR western blots 500 mM TCEPT was added to the lysate sample. Protein samples were run into NuPAGE Midi Protein gels (Thermo Fisher Scientific); 10% Bis-Tris gels were used for the detection of proteins up to 100 kDa and 3–8% Tris-Acetate gels were used for higher molecular weight proteins. Few µl of Precision Plus Protein Dual Colour marker (Bio-rad) were used to track electrophoresis. Samples were electrophoresed at 150 V in either 1× MES or 1× Tris Acetate (Thermo Fisher Scientific). Following transfer, membranes were blocked and probed with primary antibodies in 5% BSA TBS, 0.1% Tween 20 overnight at 4 °C before washing and incubating with horseradish peroxidase-conjugated secondary antibodies. Loading controls were run on the same gel as the proteins under investigation. The blot was either stripped and re-probed for GAPDH (DOCK4, DOCK9, RAC1, CDC42); or the membrane was cut at the expected molecular weight, and the segments were probed separately for the proteins of interest and GAPDH (pEGFR, EGFR). Protein signals were subsequently visualised using ECL Western Blotting Substrate (Thermo Fisher Scientific). The uncropped western blots are provided in Supplementary Fig. 1.

### Adhesion Assay

hCMEC/D3 cells (1.5 ×10^4^ per well) were seeded onto 96-well plates (Corning) coated with 1% gelatin and incubated for 72 h until they reached confluency. MDA-MB-231/Brain cells (1.5 × 10^4^ per well) were then seeded onto the hCMEC/D3 monolayer and incubated at 37 °C for 15 min before each plate was washed with PBS to remove non adherent cells. The cultures were then fixed with 4% (w/v) paraformaldehyde (PFA; Sigma-Aldrich) for 10 min at room temperature, and washed with PBS before being imaged with the Incucyte® ZOOM Live-Cell Analysis System (Essen Bioscience) using a 20× objective. The cells adhering onto the monolayer were counted from the images. Each condition was performed in 3 independent experiments and the percentage of adherent cells was calculated.

### Intercalation assay

hCMEC/D3 cells (4.5 × 10^4^ per well) were seeded onto 24 well plates (Corning) coated with 1% gelatin and incubated 72 h until they reached confluency. MDA-MB-231/Brain cells (5 × 10^4^ per well) stably expressing LifeAct-GFP were then seeded onto the hCMEC/D3 monolayer and monitored by time-lapse microscopy using the Incucyte® ZOOM Live-Cell Analysis System (Essen Bioscience) using a 20× objective. Images were taken every 30 min over a period of 5 h. Intercalated cells were quantified from the timelapse movies. A cell was considered intercalated if it was no longer round and phase bright, and it was part of the monolayer. Each condition was performed in three independent experiments and the percentage of intercalated cells was calculated.

### Elongation assay and immunofluorescence

MDA-MB-231/Parental or MDA-MB-231/Brain cells (1.5 × 10^5^ per well) were seeded onto 6-well plates (Corning) coated with Poly-L-Lysine (1:40, Sigma) and serum-starved overnight. Cells were then trypsinised and resuspended in 30 ng/ml Epidermal Growth Factor (EGF) (Sigma), or 50 ng/ml Transforming Growth Factor (TGFβ) β (Peprotech), or conditioned media harvested from 50% confluent hCMEC/D3, HUVEC, or normal culture media. The cells were then seeded onto glass coverslips coated with 1% gelatin and incubated at 37 °C for 4 h. Cells were then fixed with 4% PFA for 10 min at room temperature and washed with PBS. The cells were permeabilised with 0.1% Triton-X for 5 min, and Phalloidin Alexa Fluor 594 (Invitrogen 1:500) was added for 1 hr at room temperature. After washing with PBS the coverslips were incubated with 4,6-diamidino-2-phenylindole (DAPI) (2 µg/ml) for 1 min, washed with dH_2_O, and mounted onto microscope slides using Antifade Mountant (Thermo Fisher Scientific). Coverslips were visualised on a Zeiss Apotome microscope using 10×, 20× or 40× objectives. The aspect ratio (length/width) of cells was determined using Fiji.

### Inhibitor treatment

MDA-MB-231/Brain cells were treated with the following inhibitors: Afatinib (5 μM), SD208 (0.1 μM or 1 μM), and Y27632 (5 μM) for 8 h prior to overnight serum starvation. MDA-MB-231/Brain cells (6 × 10^3^ cells per well) seeded onto glass coverslips in 12-well plates were stimulated with 30 ng/ml EGF (Sigma), or hCMEC/D3-conditioned media, or control media (MEM or E CM) in the presence or absence of inhibitors, or vehicle (DMSO). The cells were then fixed, stained, and the aspect ratio was determined using Fiji.

### Confluency assay

MDA-MB-231/Brain cells were seeded at a density of 20,000 cells per cm^2^ and imaged at 37 °C and 5% CO_2_ using the Incucyte® Zoom Live-Cell Analysis System (Essen Bioscience). For confluency curve analysis, images were acquired longitudinally at set time points and processed using the Incucyte® Zoom Confluence processing tool to quantify the increase in cellular density over background in each condition.

### In vivo intracranial growth and bioluminescence imaging

Six- to 8-wk-old female C57BL/6J mice were purchased from Charles River Laboratories, UK. MDA-MB-231/Brain cells (1 × 10^5^ in 2 µl MEM without supplements) were stereotactically injected into the striatum (2-mm right from the midline, 2-mm anterior from bregma, 3-mm deep). During surgery, animals were anesthesized with isofluorane. Mice were monitored by noninvasive bioluminescence imaging after luciferin injection using IVIS Spectrum and Living Image software (PerkinElmer). All animal procedures performed in the study were approved by the University of Leeds Animal Welfare and Ethical Review Committee (AWERB) and performed under an approved UK Home Office project license according to Home Office Regulations and the CCCR guidelines. We have complied with all relevant ethical regulations for animal use.

### In vivo intracarotid injection and quantification of extravasation

Six- to 7-wk-old female CB17- SCID mice were purchased from Charles River Laboratories, UK. MDA-MB-231/Brain cells (2 × 10^4^ in MEM without supplements) were injected into the left internal carotid artery in MEM without supplements in a total volume of 50 μl. During surgery, mice were anesthesized with isofluorane. On Day 5 post tumour cell injection, brains were harvested after perfusing anesthetised animals with 20 ml 0.9% NaCl followed by 4% Paraformaldehyde. Isolated brain tissue fixed in 4% PFA at 4 °C overnight, then incubated in 25% sucrose and 0.1 M sodium phosphate buffer at 4 °C overnight. Brains were then snap-frozen on dry ice, cut into 30-µm floating cryosections using Thermo Scientific™ Cryotome™ FSE Cryostat (Fisher Scientific), and stored in Walter’s antifreeze (30% (v/v), ethylenglycol, 30% (v/v) glycerol, and 0.5 M phosphate buffer) at −20 °C. Floating sections were washed in PBS and blocked in blocking buffer (10% FCS, 0.3% Triton-X-100 in PBS) for 1 hr at room temperature. Primary antibodies detecting GFP and CD31 diluted in blocking buffer were added and the cryosections were incubated overnight at room temperature gently shaking. After washing in PBS, the cryosections were incubated for 2 h with Alexa Fluor secondary antibodies, DAPI (2 µg/ml) was added, and the slides were mounted using anti-fade gel mount. Images were acquired with a Zeiss ApoTome microscope using ×10 and ×20 objectives. The position of the cancer cells inside or outside the brain blood vessels was determined for individual cells and 100 events were analysed per tumour. The percentage of cells in and out of blood vessels was calculated.

### Statistics and reproducibility

The data presented represent the mean of three independent experiments. Statistical significance was evaluated using an unpaired Student’s *t* test.

### Reporting summary

Further information on research design is available in the Nature Portfolio Reporting Summary linked to this article.

### Supplementary information

Description of Additional Supplementary Files

Supplementary Data 1

Supplementary Movie 1

Supplementary Movie 2

Reporting Summary

Supplementary information

## Data Availability

All data supporting the findings of this study are available in Supplementary Data 1.
