# Supplementary material for: Brain endothelial cells promote breast cancer cell extravasation to the brain via EGFR-DOCK4-RAC1 signalling
Source: Commun Biol. 2024 May 18;7:602. doi: 10.1038/s42003-024-06200-x (PMC11102446; doi:10.1038/s42003-024-06200-x)
Supplement: Supplementary file 1 — Description of Additional Supplementary Files [file 42003_2024_6200_MOESM1_ESM.pdf]

Description of Additional Supplementary Files

File Name: Supplementary Data 1
Description: The source data behind the graphs in the paper.

File name: Supplementary Movie 1
Description: Representative example of real-time assessment of MDA-MB-231/Brain cell confluency upon stable DOCK4 depletion.

File name: Supplementary Movie 2
Description: Representative example of intercalation assays of MDA-MB-231/Brain cells upon stable DOCK4 depletion.
