# Supplementary material for: Brain endothelial cells promote breast cancer cell extravasation to the brain via EGFR-DOCK4-RAC1 signalling
Source: Commun Biol. 2024 May 18;7:602. doi: 10.1038/s42003-024-06200-x (PMC11102446; doi:10.1038/s42003-024-06200-x)
Supplement: Supplementary file 5 — Reporting Summary [file 42003_2024_6200_MOESM5_ESM.pdf]

nature portfolio

Corresponding author(s): Dr Georgia Mavria

Last updated by author(s): 30/12/23

Reporting Summary

Nature Portfolio wishes to improve the reproducibility of the work that we publish. This form provides structure for consistency and transparency in reporting. For further information on Nature Portfolio policies, see our Editorial Policies and the Editorial Policy Checklist.

Statistics

For all statistical analyses, confirm that the following items are present in the figure legend, table legend, main text, or Methods section.

n/a	Confirmed	
☐	☒	The exact sample size (*n*) for each experimental group/condition, given as a discrete number and unit of measurement
☐	☒	A statement on whether measurements were taken from distinct samples or whether the same sample was measured repeatedly
☐	☒	The statistical test(s) used AND whether they are one- or two-sided<br>*Only common tests should be described solely by name; describe more complex techniques in the Methods section.*
☒	☐	A description of all covariates tested
☒	☐	A description of any assumptions or corrections, such as tests of normality and adjustment for multiple comparisons
☒	☐	A full description of the statistical parameters including central tendency (e.g. means) or other basic estimates (e.g. regression coefficient) AND variation (e.g. standard deviation) or associated estimates of uncertainty (e.g. confidence intervals)
☐	☒	For null hypothesis testing, the test statistic (e.g. *F*, *t*, *r*) with confidence intervals, effect sizes, degrees of freedom and *P* value noted<br>*Give P values as exact values whenever suitable.*
☒	☐	For Bayesian analysis, information on the choice of priors and Markov chain Monte Carlo settings
☒	☐	For hierarchical and complex designs, identification of the appropriate level for tests and full reporting of outcomes
☒	☐	Estimates of effect sizes (e.g. Cohen's *d*, Pearson's *r*), indicating how they were calculated

Our web collection on statistics for biologists contains articles on many of the points above.

Software and code

Policy information about availability of computer code

Data collection	ImageJ2 version 2.1.0
Data analysis	PRISM 9

For manuscripts utilizing custom algorithms or software that are central to the research but not yet described in published literature, software must be made available to editors and reviewers. We strongly encourage code deposition in a community repository (e.g. GitHub). See the Nature Portfolio guidelines for submitting code & software for further information.

Data

Policy information about availability of data

All manuscripts must include a data availability statement. This statement should provide the following information, where applicable:

- Accession codes, unique identifiers, or web links for publicly available datasets
- A description of any restrictions on data availability
- For clinical datasets or third party data, please ensure that the statement adheres to our policy

All data supporting the findings of this study are available within the paper. There no data currently stored in a repository.

Research involving human participants, their data, or biological material

Policy information about studies with human participants or human data. See also policy information about sex, gender (identity/presentation), and sexual orientation and race, ethnicity and racism.

Reporting on sex and gender	N/A
Reporting on race, ethnicity, or other socially relevant groupings	N/A
Population characteristics	N/A
Recruitment	N/A
Ethics oversight	N/A

Note that full information on the approval of the study protocol must also be provided in the manuscript.

Field-specific reporting

Please select the one below that is the best fit for your research. If you are not sure, read the appropriate sections before making your selection.

☒ Life sciences ☐ Behavioural & social sciences ☐ Ecological, evolutionary & environmental sciences

For a reference copy of the document with all sections, see nature.com/documents/nr-reporting-summary-flat.pdf

Life sciences study design

All studies must disclose on these points even when the disclosure is negative.

Sample size	Sample sizes for the in vivo experiments were based on previously published protocols by Lorger et al. Am J Pathol 176, 2958-2971 (2010). The sample sizes for cell shape quantifications were based on pilot experiments with control cells, or cell with DOCK4 knockdown, that indicated that statistically significant differences were obtained when 20 or more cells were analysed per condition.
Data exclusions	No data exclusions have been made.
Replication	All experiments were independently replicated at least three times and the data were included in the analyses.
Randomization	Randomization was not applicable when testing cell shape and the number of cancer cells extravasated from the circulation into the brain under conditions of knockdown of DOCK4 or its interaction partners due to the specific nature of the experimental design. The experimental groups were intentionally designed to represent distinct genetic manipulations, making random assignment unnecessary. Instead, careful consideration of the experimental design and appropriate controls were used to address potential sources of bias and variability in the experimental results.
Blinding	Cell shape quantifications were performed a blinded manner so that the identity of samples was unknown at the time of data collection.

Reporting for specific materials, systems and methods

We require information from authors about some types of materials, experimental systems and methods used in many studies. Here, indicate whether each material, system or method listed is relevant to your study. If you are not sure if a list item applies to your research, read the appropriate section before selecting a response.

Materials & experimental systems

n/a	Involved in the study	
☐	☒	Antibodies
☐	☒	Eukaryotic cell lines
☒	☐	Palaeontology and archaeology
☐	☒	Animals and other organisms
☒	☐	Clinical data
☒	☐	Dual use research of concern
☒	☐	Plants

Methods

n/a	Involved in the study	
☒	☐	ChIP-seq
☒	☐	Flow cytometry
☒	☐	MRI-based neuroimaging

Antibodies

Antibodies used	Abcam, anti-GFP (ab2090, 1:1000); BD Biosciences, anti-CD31 (clone MEC 13.3, 1:50); Bethyl Laboratories, anti-DOCK4 (A302-263A, 1:1,000); Cell signalling, anti-EGFR (D38B1, 1:500), anti-pEGFR (D7A5, 1:500); Millipore, anti-RAC1 (clone 23A8, 1:1,000); Proteintech, anti-GAPDH (60004, 1:1,000), anti-DOCK9 (18987; 1:1,000); Santa Cruz, anti-CDC42 (sc-8401, 1:50); Thermo Fisher Scientific, Alexa Fluor secondary antibodies 488 and 549 (1:200).
Validation	All antibodies used have been employed in multiple published studies. The antibody against pEGFR was validated through stimulation of pEGFR with EGF. The specificity of the antibodies against DOCK4, DOCK9, RAC1, and CDC42 was confirmed through siRNA-mediated knockdown.

Eukaryotic cell lines

Policy information about cell lines and Sex and Gender in Research

Cell line source(s)	MDA-MB-231/Brain, hCMEC/D3
Authentication	The cell line MDA-MB-231/Brain was obtained by Dr Mihaela Lorger {Lorger et al. Am J Pathol 176, 2958-2971 (2010)]. Its authenticity was confirmed through morphological characteristics and propensity to metastasise to the brain when injected into the circulation. The parental cell line, also obtained by Dr Lorger, was used as control in some of the experiments. The hCMEC/D3 cell line was purchased from MERCK (https://www.merckmillipore.com/GB/en/product/Blood-Brain-Barrier-hCMEC-D3-Cell-Line,MM_NF-SCC066) and its authenticity was confirmed by the expression of the endothelial cell marker VE-cadherin.
Mycoplasma contamination	The cell lines tested negative for mycoplasma contamination.
Commonly misidentified lines (See ICLAC register)	No commonly misidentified cell lines were utilised in the study.

Animals and other research organisms

Policy information about studies involving animals; ARRIVE guidelines recommended for reporting animal research, and Sex and Gender in Research

Laboratory animals	Six- to 7-wk-old female CB17/SCID mice were purchased from Charles River Laboratories, UK.
Wild animals	N/A
Reporting on sex	Female-only mice were sued for the following reasons:<br>The use of female-only mice in breast cancer research experiments is often justified for several reasons:<br><br>1. Relevance to human breast cancer: Breast cancer primarily affects women, and there are significant sex differences in the incidence, progression, and response to treatment of breast cancer. By using female-only mice, we can better model the disease in the population it primarily affects, thereby improving the relevance and translational potential of findings to human breast cancer.<br><br>2. Hormonal influences: Hormones, particularly estrogen and progesterone, play a significant role in the development and progression of breast cancer. Using female-only mice allows us to study the specific effects of these hormones on breast cancer development and progression without the complicating factor of male hormones.<br><br>3. Reduction of variability: By using only female mice, we can reduce the variability in experimental results that might arise from differences in hormone levels, reproductive cycles, and other sex-specific factors. This can help to improve the statistical power of studies and the ability to draw meaningful conclusions.<br><br>4. Ethical considerations: Limiting the use of animals in research is an important ethical consideration. By focusing on female mice for breast cancer research, we can reduce the overall number of animals used in experiments while still obtaining valuable data relevant to the disease.
Field-collected samples	N/A
Ethics oversight	All animal procedures performed in the study were approved by the University of Leeds Animal Welfare and Ethical Review Committee (AWERB) and performed under an approved UK Home Office project license according to Home Office Regulations and the CCCR guidelines.

Note that full information on the approval of the study protocol must also be provided in the manuscript.

Plants

Seed stocks	N/A
Novel plant genotypes	N/A
Authentication	N/A
