# Supplementary material for: Brain endothelial cells promote breast cancer cell extravasation to the brain via EGFR-DOCK4-RAC1 signalling
Source: Commun Biol. 2024 May 18;7:602. doi: 10.1038/s42003-024-06200-x (PMC11102446; doi:10.1038/s42003-024-06200-x)

Supplementary information

Supplementary Figure 1. Uncropped western blots. Yellow marking denotes the re-probed area for GAPDH.

Fig. 1a

DOCK4 (lanes 2-4)

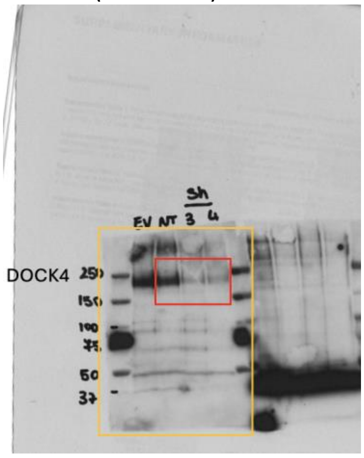

GAPDH

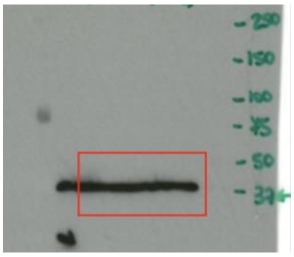

Fig. 2f

Rac1 (lanes 1-3)

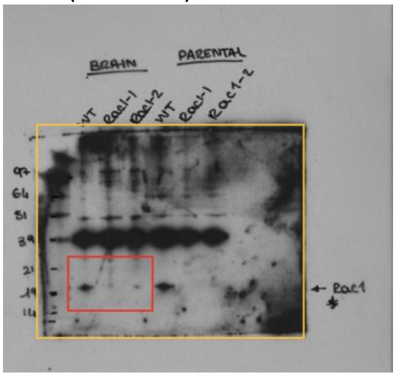

GAPDH

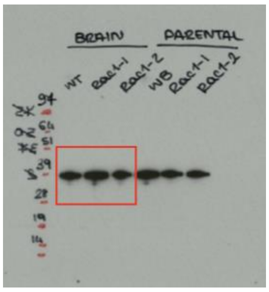

Fig. 4a

pEGFR (lanes 1,2,3,4,5,6,9,10)

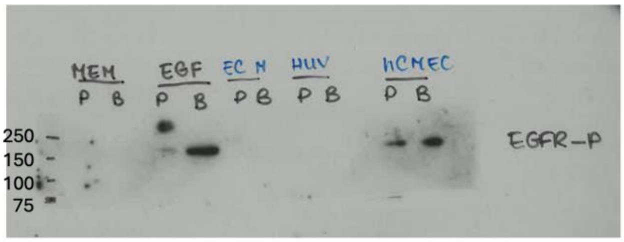

GAPDH (pEGFR)

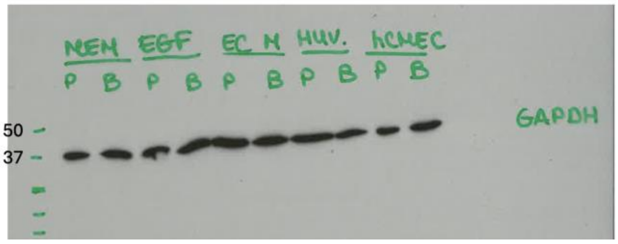

EGFR (lanes 1,2,3,4,5,6,9,10)

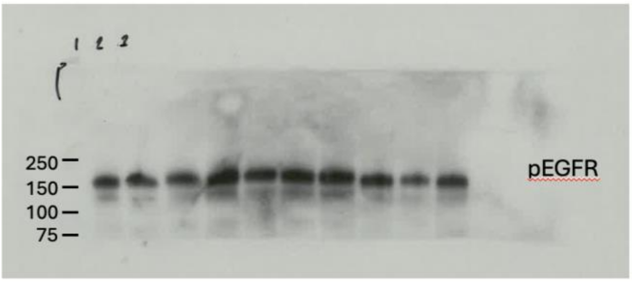

GAPDH (EGFR)

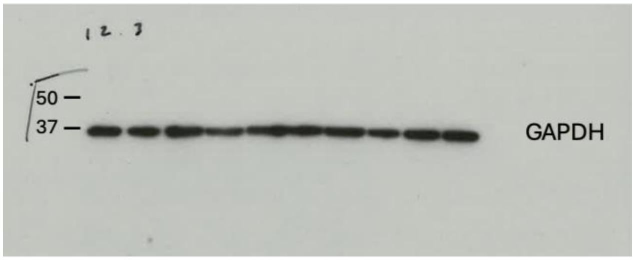

Fig. 4c

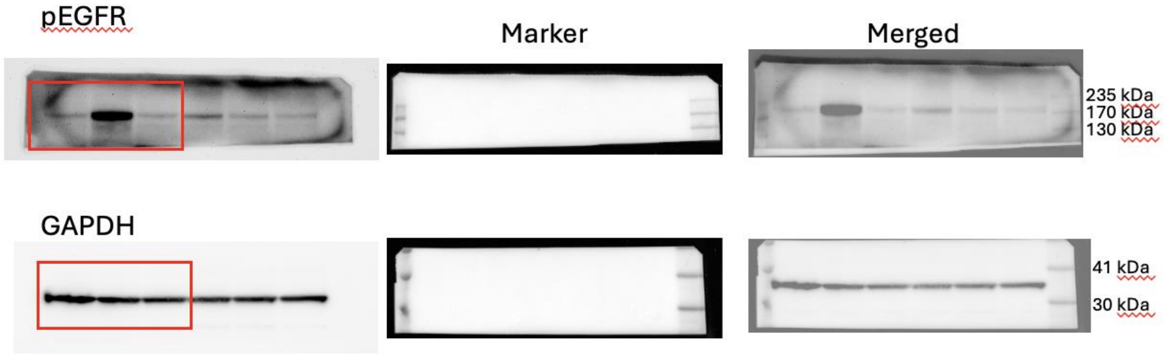

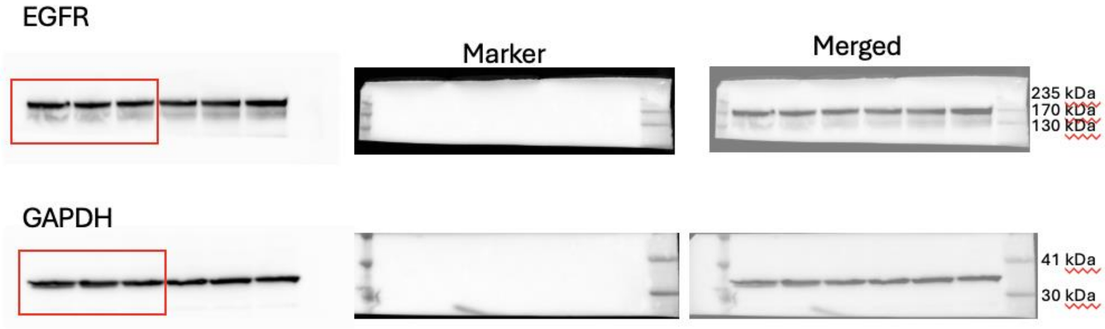

Fig. 5b

DOCK9

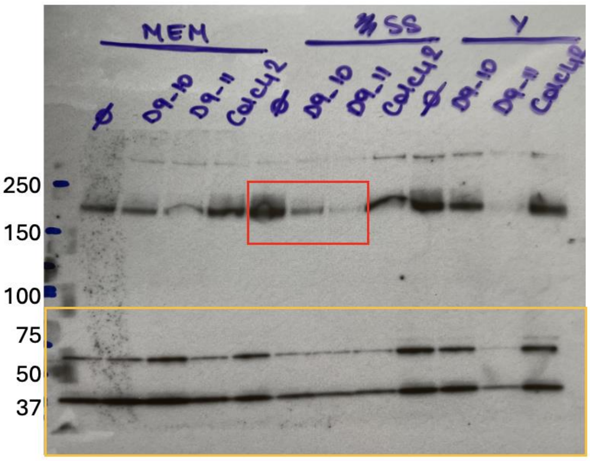

GAPDH

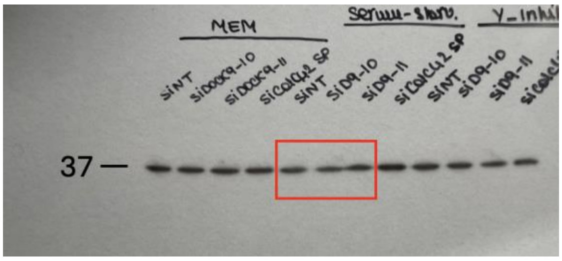

Fig. 5c

CDC42

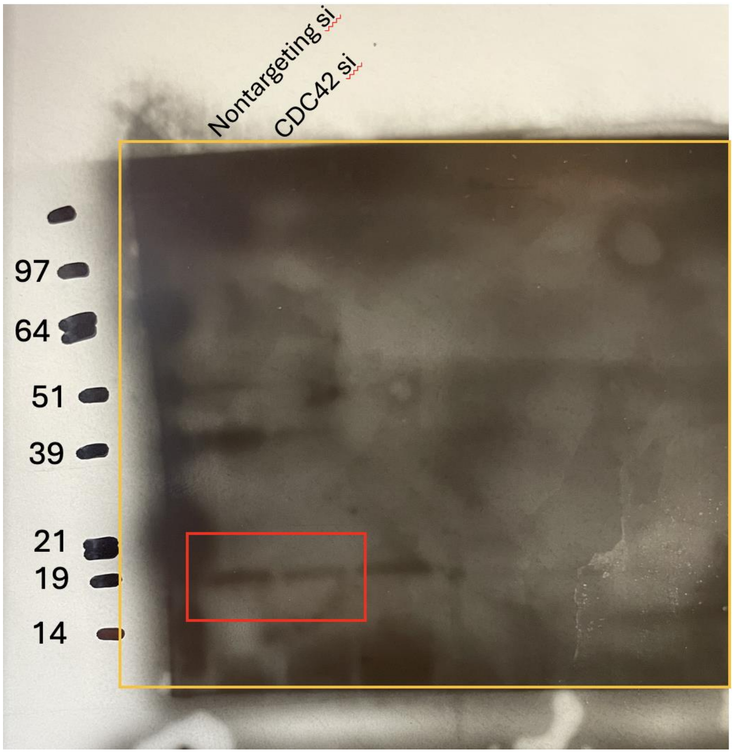

GAPDH

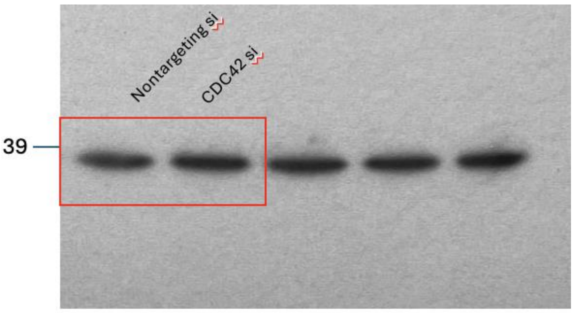

Supplement: Supplementary file 6 — Supplementary information [file 42003_2024_6200_MOESM6_ESM.pdf]
